# “Anti-Condensation” Aluminum Superhydrophobic Surface by Smaller Nanostructures

**DOI:** 10.3389/fbioe.2022.887902

**Published:** 2022-04-26

**Authors:** Kangning Li, Ying Zhao, Jintao Yang, Jie Feng

**Affiliations:** ^1^ College of Materials Science and Engineering, Zhejiang University of Technology, Hangzhou, China; ^2^ Jinhua Polytechnic, Jinhua, China

**Keywords:** aluminum, superhydrophobic surface, anti-condensation, microscopic mechanism, smaller nanostructures

## Abstract

According to classical heterogeneous nucleation theory, the free energy barrier (ΔG_c_) of heterogeneous nucleation of vapor condensation ascends dramatically as the substrate nanostructure diameter (R_s_) decreases. Based on this idea, we fabricated two types of superhydrophobic surfaces (SHSs) on an aluminum substrate by different roughening processes and the same fluorization treatment. Water vapor condensation trials by optical microscope and ESEM confirmed that on SHSs with submicron rectangle structures, a typical self-propelled motion of condensates or jumping condensation occurred. However, on SHS with coral-like micro/nano-structures, vapor nucleation occurred tardily, randomly, and sparsely, and the subsequent condensation preferentially occurred on the nuclei formed earlier, e.g., the condensation on such SHS typically followed the Matthew effect. Higher vapor-liquid nucleation energy barrier caused by smaller fluorinated nanostructures should be responsible for such a unique “anti-condensation” property. This study would be helpful in designing new SHSs and moving their application in anti-icing, anti-fogging, air humidity control, and so on.

## Introduction

Many plants exhibit remarkable water repellency owing to their rough surface. The textured surface traps air underneath water droplets and the air cushioning gives rise to superhydrophobicity (Barthlott and Neinhuis, 1997; Neinhuis and Barthlott, 1997). However, biomimetic superhydrophobic surfaces (SHSs) generally do not retain water repellency when exposed to a condensing environment (Zhao and Yang, 2017; Chen et al., 2018; Zhao et al., 2018; Orejon et al., 2019). Water condensates proceeding from nanoscale nuclei tend to penetrate into the surface texture and displace the trapped air, forfeiting the superhydrophobicity. Along with the condensation proceeds, arrays of visible, glittering, transparent, and adhesive large Wenzel drops (3–5 mm in diameter) cover the SHSs gradually. This seriously limits their applications in sustained dropwise condensation (Hao et al., 2018; Wang et al., 2020), water collection (Zheng et al., 2010), anti-icing (Kreder et al., 2016; Caldona et al., 2017; Zhu et al., 2020), and anti-corrosion (Xue et al., 2020). In some cases, these SHSs even represent a worse performance than general hydrophobic surfaces do such as increasing ice adhesion strength once the ice forms (Kreder et al., 2016; Caldona et al., 2017).

Recently, the self-propelled motion of condensate drops on some SHSs has attracted increasing attention due to its potential applications in delaying frost growth (Hao et al., 2014; Jiang et al., 2020; Mohammadian et al., 2020), enhancing condensation heat transfer (Hao et al., 2018; Sarode et al., 2020), stronger self-cleaning (Geyer et al., 2020), and breathable anti-condensation coating on buildings (Wu et al., 2021). Wang et al. (2021) demonstrated that the vapor molecules can be intercepted by oblique nanowires and preferentially nucleate at near-surface locations, avoiding the penetration of vapor into the microscale gaps.

In our earlier studies (Feng et al., 2012a; Feng et al., 2012b), we have confirmed that nuclei formed within the nanogaps of SHSs would grow and coalesce into micro-droplets. Then the micro-droplets derive themselves upwards and form into Cassie droplets. It is such a Cassie state that causes the spontaneous motion of drops after coalescence. A nanostructure with sufficiently narrow spacing and high perpendicularity is favorable to form such a Cassie condensation. According to classical heterogeneous nucleation theory (Liu, 1999), the free energy barrier (ΔG_c_) of heterogeneous nucleation of vapor condensation ascends dramatically as the substrate nanostructure’s diameter (R_s_) decreases. No nucleation would bring none condensation. Based on this principle, new types of SHS with a more obvious anti-condensation property may be created by designing fine nanostructures.

In this study, we fabricated two types of SHSs on an aluminum substrate by two different roughening processes and the same fluorization treatment. One was only by HCl etching and the other was by HCl etching and by further immersing in hot water. Water vapor condensation trials confirmed that although both two surfaces were superhydrophobic and supported Cassie condensation, only SHS by HCl etching and further by hot water treatment showed an obvious anti-condensation property, e.g., the condensate droplets appeared tardily, randomly, and sparsely on it. Most of the SHS areas appeared dry. A much higher nucleation energy barrier caused by much smaller nanostructures should be responsible for such phenomena. This study opens a new door for designing new SHSs and moving their applications in fields such as anti-icing, anti-fogging, anti-corrosion, and air humidity control.

## Experimental Section

### Superhydrophobic Surfaces Preparation

The aluminum foils with size of 6 cm × 5 cm × 0.5 mm (purity 99.99%) were ultrasonically washed in acetone and ethanol to get rid of organic contamination. The cleaned aluminum foils were etched in 9 wt% HCl aqueous solution for 12 min at room temperature. After being rinsed with deionized water, a part of the samples were further immersed in deionized water (50°C) for 40 min and subsequently dried with nitrogen. Then the two batches of samples were incubated in a 0.5 wt% hexane solution of 1H, 1H, 2H, and 2H-perfluorodecyltriethoxysilane (FAS17, Sigma) at room temperature for 1 h, followed by drying at 120°C for 1 h.

### Morphology and Wettability of the Superhydrophobic Surfaces

The morphologies of as-prepared aluminum surfaces were characterized by field emission scanning electron microscopy (FE-SEM, S4700, Hitachi, Japan). For each surface, its nanostructure parameters such as the diameter or width/length and gap space were measured and calculated statistically from the SEM images. The water contact angles (CAs) and slide angles (SAs) were measured by using a Dataphysics OCA35 contact-angle system with a temperature control stage. This stage can precisely maintain the temperature of SHS from -30–160°C. The volume of the water droplet used for the CA measurements is 4 μL. The CAs were obtained by averaging five measurement results.

### Condensation Under Ambient Condition

Condensation experiments were performed in a closed room with an area of 25 m^2^ and a height of 3 m. The ambient temperature was controlled at 28 ± 1°C and the relative humidity (RH) was adjusted at 80 ± 2%. The surface superhydrophobilized aluminum foils with a size of 3 cm × 3 cm × 0.5 mm were placed on a horizontally orientated Peltier cooling stage with the hot side cooled by recirculating water. The sample surface was maintained at 0–1°C. The spontaneous motion of condensate droplets was observed and visualized by an optical microscope (Nikon LV 150) with a ×10 objective and charge-coupled device camera (CCD) at 25 fps. The phenomena were quantified by analyzing 2 min representative videos. Four short periods of time (only 1 s) spacing 30 s, all together 5 × 25 pieces of snapshots were used to quantify the average numbers of distinguishable drop location changes in 1 s videos (here named as “spontaneous motion frequency”) (Feng et al., 2012a; Feng et al., 2012b).

### Condensation Dynamics in ESEM

The microscale dynamics of vapor condensation on the sample surfaces were *in situ* visualized using an environmental scanning electron microscopy (ESEM, FEI Quanta 200 FEG) with a Peltier cooling stage. The sample was placed on a stainless steel sample holder that was rested on the Peltier cooling stage. The drop condensation was imaged using a gaseous secondary electron detector. The electron beam voltage was set at ∼30 keV in order to ensure better contrast for image visualization. The condensation process can be triggered by precisely controlling the stage temperature and the water vapor pressure in the chamber. In this experiment, the temperature of the Peltier cooling stage was fixed at ∼1°C. The vapor pressure was gradually increased to ∼800 Pa, at which the vapor started to nucleate on the sample surface, and then maintained at ∼800 Pa during imaging. The images were taken every 1.6 s.

## Results and Discussion

### Morphology and Superhydrophobicity of as-Fabricated Surfaces

**FIGURE 1:**
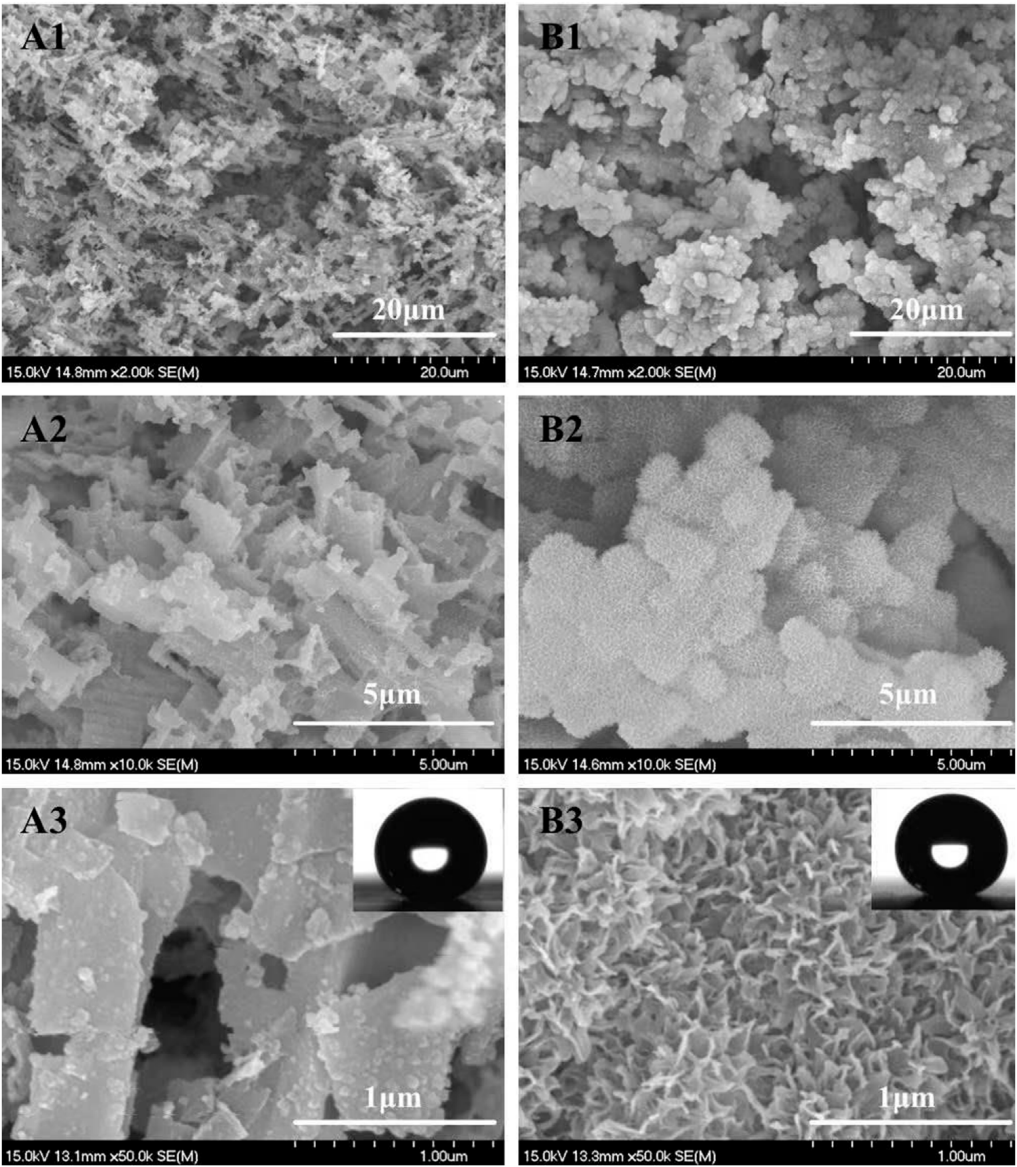
FESEM images of the textured aluminum surface obtained by 12 min HCl etching (9 wt%) at 20°C **(A)** and further immersing in 50°C water for 40 min **(B)**. Magnification from A1 to A3 or B1 to B3 is increased. The insets were profiles of 4 μL water droplets showing WCA both at ∼155°.

Similar to the results of Yin et al. (2012) and Z. Zhang Yang et al. (2011), rectangle-shaped submicron-structure (Figure 1A1–3) and coral-like micro/nano-hierarchical structures (Figure 1B1–3) were obtained on the aluminum surface after HCl etching and HCl etching combined with hot water treatment, respectively. Vulnerable dislocation sites inside the crystalline aluminum should be responsible for such a submicron rectangle structure (∼0.5–1 μm) (Yin et al., 2012). While the reaction of aluminum with hot water starting from the dissolution of aluminum and followed by the deposition of aluminum hydroxide colloidal particles on the aluminum surface should be responsible for the coral like micro/nano-hierarchical structures (He et al., 2012). The average width of nano-flakes is ∼10 nm and the average space is ∼100 nm (Figure 1B3).

CAs and SAs measurement showed that the sessile CAs of two types of as-prepared surfaces were both larger than 150° (Figure 1, insets) and the SAs were both less than 2°. This demonstrated that both two types of surfaces were typical superhydrophobic and the intrinsic surface energy was sufficiently low. The latter is one of two key factors affecting the vapor condensation nucleation energy barrier (Liu, 1999). Compared with the anodization method, simple hot water immersing supplied a facile process in creating dense nanostructures and narrow nanogaps on the aluminum substrate, which is necessary for forming larger upward Laplace pressure to the droplets condensed within the gaps (if they could form there) and thus bringing Cassie condensation and rapid self-propelled motion phenomenon to condensate droplets (Feng et al., 2012a; Feng et al., 2012b).

### Condensation Under Ambient Condition

**FIGURE 2:**
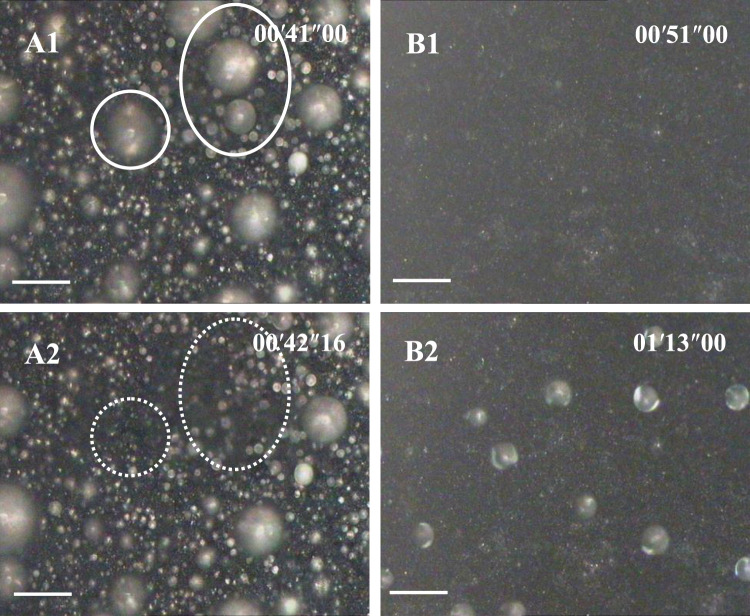
Water vapor condensation behavior on SHS with submicron rectangle microstructures **(A)** series (implying Cassie or jumping condensation), and on SHS with coral- like micro/nano-structures **(B)** series, implying an anti-condensation character). The scale bar is 60 μm. The temperature of SHSs was 0–1°C. The environmental RH was 80 ± 2% (28 ± 1°C). The time scale in the images is minute, second, and millisecond.Supplementary Video S1,S2 corresponding to A and B are available in the Supporting Information.

Figure 2 shows the time-lapse top-view optical images of dropwise condensation on aluminum surfaces prepared by two different etching methods. It clearly demonstrates that different surface structures do bring different condensation behaviors. On a rectangle submicron structured SHS, condensate droplets appeared in a classical self-propelled motion or “jumping” behavior, e g., condensation, is continuously, covering all areas and homogeneous (Figure 2A). The spontaneous motion frequency began at the high level (>100 drops/s), changed a little in 1 min and then gradually decreased, and finally balanced at 70 drops/s. Re-nucleation and growth of condensate droplets appeared on any region of the SHS especially including bare areas caused by droplet move-away. However, on coral-like micro/nano-structured SHS, condensate droplets appeared slowly (∼50 s delay), dispersedly, and sparsely in the whole condensation procedure. Most of SHS was always bare and dry. Primary nucleation occurred randomly and the subsequent nucleation occurred preferentially on the droplets formed by these former nuclei. Because the distance between the droplets was so far, the coalescence opportunity was so low that no self-propelled motion or “jumping” appeared throughout the condensation process (Figure 2B).

### Condensation in ESEM

**FIGURE 3:**
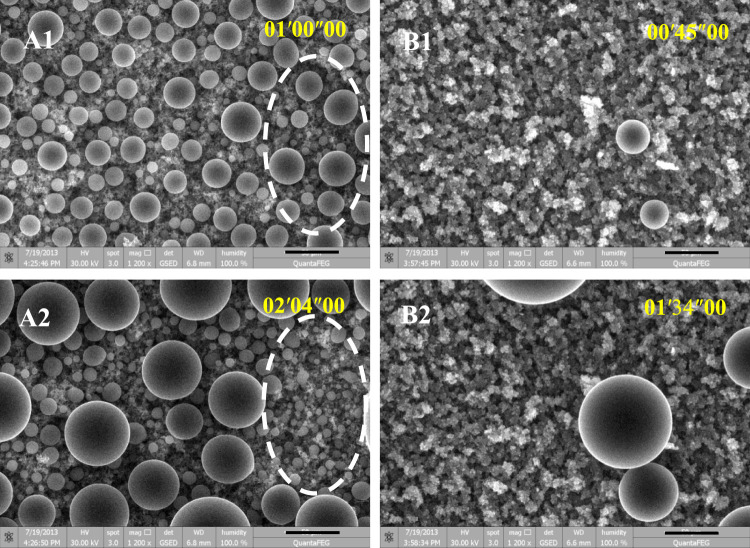
Time-lapse ESEM images of vapor condensation on SHSs with submicron rectangle structures (A1-2) and with coral-like micro/nano-structures (B1-2), respectively. The scale bar is 50 μm. The temperature of the sample stage was fixed at ∼1°C. The vapor pressure was gradually increased to ∼800 Pa, at which the vapor started to nucleate on the sample surface, and then maintained at ∼800 Pa during imaging. The images were taken every 1.6 s. The time scale in the images is minute, second, and millisecond.

To better understand the aforementioned preferential condensation phenomenon, we further apply ESEM to observe the condensation dynamics on both types of SHSs. Figure 3 shows time-lapse images of condensation on the SHSs with submicron rectangle microstructures and coral like micro/nano-structures, respectively. It clearly proved the results of the microscopy video: both SHSs presented spherical Cassie state condensate droplets, however, only SHS with coral-like micro/nano-structures appeared to have an obvious anti-condensation property. That is, on SHSs with submicron rectangle microstructures, spherical droplets emerged continuously and coalesced successively thus forming droplets with dispersed diameters (Figure 3A). However, on SHS with coral-like micro/nano-structures, vapor nucleation occurred tardily, randomly, and sparsely, and the subsequent condensation preferentially occurred on these earlier formed nuclei. In the microscopy visual field, only several large drops grew up through their growth and asymmetric coalescence, while most areas were always dry (Figure 3B).

### Anti-Condensation Mechanism Analysis

As it showed in Figures 2B, 3B, when homogeneous dropwise condensation continuously occurred on SHS with submicron rectangle microstructures, no condensate droplets appeared on most districts of SHS with coral-like micro/nano-structures. This phenomenon can be explained by the classical nucleation theory. Essentially, vapor condensation at least includes the process of nucleation and growth. Nucleation is the process of vapor molecules clustering together. It was generally triggered by supersaturation and with or without preferential sites such as dust and surface nanostructures (so called homogeneous or heterogeneous nucleation). Critical nucleation radius is the minimum size that must be formed by vapor molecules clustering before a droplet is stable and begins to grow. It mainly depends on supersaturation caused by dew point, supercooling temperature, and RH. According to the classical nucleation theory (Liu, 1999), the radius of the critical nucleus (r_c_) in vapor condensation can be estimated from:

$$RT\;\mathrm{In}\frac{P_{r}}{P}=-\frac{2\gamma V_{m}}{r}$$

Where R is ideal gas constant (8.314 J⋅mol^−1^K^−1^), T is the temperature of condensation (273.15 K), $P_{r}$ is the vapor pressure over a curved interface of a droplet with radius r, *P* is the equilibrium vapor pressure above a flat surface of the condensed phase at *T* (*P* is 0.61129 kPa at 0°C), *γ* ≈ 7.56 × 10^–2^ J/m^2^ is the water (0°C) interfacial tension, and *ν* ≈ 1.8 × 10^–5^ m^3^/mol is the water molar volume. When the $P_{r}$ ≤ P (28°C) (3.7818 kPa) × 80 % (RH at 28°C), nucleation occurs and the corresponding radius is at an equilibrium or critical radius (here named $r_{c}$ , which is 0.75 nm after calculation).

The critical radius is the minimum droplet radius for the formation of stable nuclei. However, it is a concept suitable to homogeneous nucleation. In most cases, nucleation occurs at nucleation sites on surfaces contacting the vapor, and thus results in heterogeneous nucleation. Comparing with critical radius, the free energy barrier of nucleation is another index being developed to describe the difficulty of nucleation especially those that occur on the surfaces with nano or micro-structures (heterogeneous nucleation). According to the classical heterogeneous nucleation theory (Liu, 1999), the effect of the surface structure on the free energy barrier of heterogeneous formation of condensate droplet ( $\text{Δ}G_{c}$ ) can be readily estimated as:

$$\text{Δ}G_{c}=\text{Δ}G_{c}^{\mathrm{hom}o}f\left(m,x\right)$$

Where ΔG_c_^homo^ is the free energy barrier forming a droplet in a homogeneous way, f(m,x) is the ratio of free energy barrier for nucleation around a spherical particle relative to that in the bulk, e.g., a factor that reduces the energy barrier of heterogeneous nucleation. m = cosθ_flat_ with θ_flat_ = 108° for FAS treatment (Feng et al., 2012a), and x = R_s_/r_c_. Since r_c_ is certain (0.75 nm), f(m,x) only changes with the radius of nucleating substrate structures (R_s_). As Liu et al. (1999) derived, the f(m,x) ascends dramatically as the nanostructure diameter decreases till it approaches 1. When the condensation conditions (RH, supercooling, et al.) are same, the smaller nanostructure on SHS would bring a more difficult nucleation.

On the SHS with coral-like micro/nano-structures (Figure 1B), the average width of the nano-flakes is ∼10 nm and the average space is ∼100 nm. This means that the corresponding apparent radius of nucleating structures (R_s_) are ∼5 nm and ∼50 nm, respectively, both larger than the critical nucleus r_c_ (0.75 nm). However, on SHS with submicron rectangle structures, the average apparent radius of nucleating structures (R_s_) are ∼0.5–1 μm, which is much larger than the critical nucleus r_c_ (0.75 nm). This means that the $\Delta G_{c}$ on the submicron rectangle structures should be lower than that on the coral-like micro/nano-structures. A similar result had also been obtained by Lo et al. (2014) , where they found that water vapor preferentially condenses on the designed microgrooves on the Si nanowire surface (both with CA∼145°).

**FIGURE 4:**
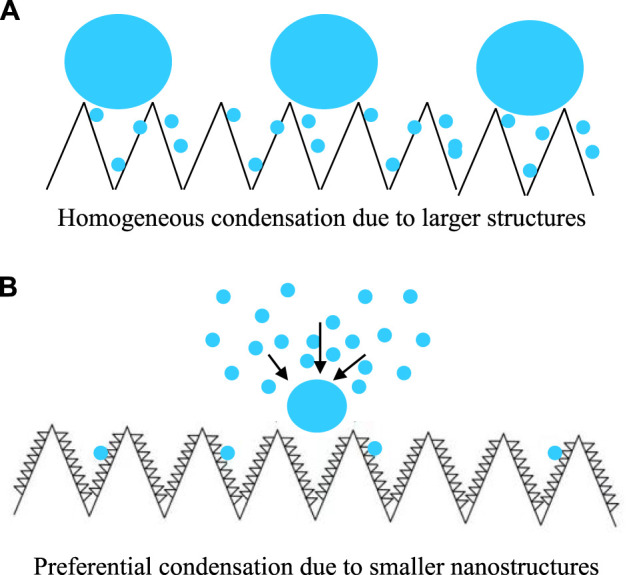
Scheme of water vapor condensation on SHS with submicron rectangle structures **(A)**, condensate droplets appeared immediately, continuously, and densely, and on the SHS with much smaller nanostructures **(B)**, condensate droplets appeared tardily, randomly, and sparsely, and the subsequent condensation preferentially occurred on the nuclei formed earlier, respectively.

The structure of the SHS has a strong effect on the nucleation rate *J via* the inverse exponential dependence on $\text{Δ}G_{c}$ , $J=J_{0}\exp\left(-\text{Δ}G/kT\right)$ (Varanasi et al., 2009). As a result, on the SHS with coral-like micro/nano-structures, due to higher vapor–liquid nucleation energy barrier caused by the finer nano-structures, vapor nucleation is difficult (∼50 s delaying) comparing with that on SHS with submicron rectangle structures (immediately, continuously, and densely). However, difficulty does not mean impossible. Vapor nucleation may also occur at some gaps of the surfaces. Once the primary nucleation is completed on the SHS with coral- like micro/nano-structures, the subsequent nucleation preferentially occurs on these primary nuclei (Figure 4 This is because they are hydrophilic, which dramatically decreases the $\Delta G_{c}$ (Liu, 1999; Varanasi et al., 2009). As a result, the condensation occurred tardily, randomly, and sparsely. The condensation proceeded along the typical Matthew effect, e.g., always occurred on special sites (primary nuclei or defects). If we could fabricate SHS with homogeneous hydrophilic nano sites, a new type of condensation would be expected (Xing et al., 2020).

## Conclusion

In summary, we found an interesting phenomenon, e.g., “Matthew effect condensation” or “anti-condensation”, on SHS with much smaller nanostructures. Different to the classical self-propelled motion or “jumping” behavior of condensate droplets on SHS with relatively larger structures, condensate droplets on SHS with smaller nanoflakes appear slowly (∼50 s delay), dispersedly, and sparsely in the whole condensation procedure. Condensation started from random nucleation and the subsequent nucleation preferentially occurred on these primary nuclei. As a result, most of the SHS area appears dry during the condensation procedure. A much higher vapor–liquid nucleation energy barrier caused by much smaller nanostructures should be responsible for such a unique “anti-condensation” property. This study would be helpful in designing new SHSs and moving their application in anti-icing, anti-fogging, air humidity control, and other relative fields.

## Data Availability

The original contributions presented in the study are included in the article/Supplementary Material, further inquiries can be directed to the corresponding author.
